# Prophylactic effect of intranasal oxytocin on brain damage and neurological disorders in global cerebral ischemia in mice

**DOI:** 10.22038/ijbms.2020.50265.11456

**Published:** 2021-01

**Authors:** Marzieh Saffari, Shahein Momenabadi, Abbas Ali Vafaei, Abedin Vakili, Mahdi Zahedi-Khorasani

**Affiliations:** 1 Research Center of Physiology, Semnan University of Medical Sciences, Semnan, Iran; 2 Department of Physiology, Faculty of Medicine, Semnan University of Medical Sciences, Semnan, Iran

**Keywords:** Apoptosis, Interleukin-1β, Oxytocin, Stroke, Tumor necrosis factor

## Abstract

**Objective(s):**

A few experimental studies have shown the therapeutic effects of oxytocin on focal cerebral ischemia. In this study, the prophylactic effect of intranasal oxytocin on brain damage was investigated in a cerebral ischemic model.

**Materials and Methods:**

Intranasal oxytocin (8 IU/per mouse) was prescribed daily for one week. Cerebral ischemia was performed through bilateral common carotid artery occlusion (BCCAO) for 20 min and then blood flow was restored for 24 hr. Finally, neurological disorders, spatial learning and memory, neuronal death, and neuronal apoptosis were assessed in CA1, CA3, and dentate gyrus. Also, levels of interleukin-1β (IL-1β) and Tumor necrosis factor-alpha (TNFα) were measured in the hippocampus.

**Results:**

Induction of global ischemia leads to neurological disorders and impairment of spatial learning and memory that are improved by pre-treatment with oxytocin (*P*<0.01).

Cresyl violet staining showed that pretreatment with oxytocin significantly reduced the number of dead nerve cells in CA1, CA3, and dentate gyrus by 40.7, 32, and 34.3%, respectively. Also, positive TUNEL cells in CA1, CA3, and dental gyrus decreased by 15, 30, and 27%, respectively. In addition, levels of TNFα and IL-1β, which were extensively increased in ischemic mice, were significantly reduced with oxytocin pre-treatment.

**Conclusion:**

Pre-treatment of oxytocin reduces ischemic damage and improves neurological function and spatial memory. The neuroprotective effect of oxytocin is mediated by a decrease in cell death, apoptosis, and inflammatory mediators TNFα and IL-1β. Pre-treatment with oxytocin may be useful in people who are prone to stroke.

## Introduction

Stroke and ischemia-reperfusion injury are some of the leading causes of death and illness in developed countries, so research is critical to finding ways to prevent and treat the disease.

Neuropeptides are one of the choices in this regard. They are the largest group of neurotransmitters in the central nervous system that regulate cellular and physiological functions in the nervous and endocrine systems (1). The rate of the disease varies between male and female, apparently due to sex hormones, and estrogen has protective effects in cerebral ischemia. For example, the gastric secretory responses after brain injury and the consequences of ischemic renal preconditioning differ between the sexes of rats (2-4). Also, the rate of stroke is about 30% higher in men compared with women (5). Estrogen set-out the production of oxytocin in various organs like the brain. Besides interleukin (IL)-1β, IL-6, interferon τ, and oxytocin control the production of oxytocin receptors (6). Oxytocin responds to some stressors and modulates a lot of physiological activities in nervous and other systems (7). For instance, oxytocin protects against inflammation and oxidative stress caused by toxic drugs or ischemia. Renal damage due to cisplatin toxicity is prevented to a great extent by the anti-inflammatory effect of oxytocin (8). Pretreatment with oxytocin increases cell viability and reduces oxidative stress-induced cell damage in primary rat neurons that have undergone oxygen-glucose deprivation/reperfusion. However, oxytocin treatment was ineffective during a stroke. Oxytocin modulates GABA-A receptor levels, which reduce the excitability of neurons by reducing the entry of chlorine into the cell (9). Oxytocin’s neuroprotective potential was also assessed in a transient middle cerebral artery occlusion (tMCAO) model. Oxytocin reduces the volume of the infarction in the cerebral cortex and striatum by decreasing calpain-1 and apoptosis of neurons in the penumbra region of ischemia (10). 

To better understand the role of oxytocin in stroke, the study examined the prophylaxis effect of intranasal oxytocin on neuronal damage and apoptosis, neurological function, spatial learning and memory, and inflammatory mediator of TNFα and IL-1β in the general ischemic model in mice.

## Materials and Methods

***Animals***

Adult male albino mice (35–40 g, 2–4 months old) were obtained from the Pasteur Research Institute in Tehran. All research was conducted in accordance with ethical guidelines for laboratory animals. The research protocol was approved by SUMS Research Ethics Committee (ethical code number: IR.SEMUMS.REC.1398.69).

***Experimental protocols and groups***

The effect of intranasal oxytocin (oxt) administration on neurological function, spatial memory, neuronal damage, apoptosis, TNFα, and IL-1β in ischemic mice was investigated. Therefore, 36 mice were randomly divided into 6 equal groups (6 mice in each group): Sham-operated (sham), ischemic, and oxt groups.

Oxytocin (Sigma-Aldrich, Germany) at a dose of 8 IU/per mouse or saline as vehicle (10 µl), was administered daily by a catheter (PE-10) for 30 sec into the bilateral nostrils for a week. Subsequently, under chloral hydrate anesthesia (400 mg/kg IP), cerebral ischemia was induced by bilateral common carotid artery occlusion (BCCAO) for 20 min and blood flow was restored for 24 hr. Neurological disorders and spatial learning and memory were evaluated and then the animals were sacrificed for the next experiments.

***Evaluation of neurological disorders and spatial memory***

To assess motor and sensory performance, an adjusted neurological severity score was used. Briefly for motor function test: the mice were hung by the tail, and the flexion of the hind or front limbs and the bending of the head were recorded within 30 sec. The mice were also placed on the ground and the inability to walk straight, turning towards, or falling to one side was recorded. Also, the absence of the pinna reflex (a head shake when touching the auditory meatus), the absence of corneal reflex (an eye blink when lightly touching the cornea with cotton) were used to assess sensory function (11). The neurological disorder score includes 10–14 severe injuries, 5–9 moderate injuries, and 1–4 minor injuries. The neural test was performed by a blinded researcher (12). 

Radial Arm Water Maze (RAWM) was used for spatial learning and memory assessment (13). The mice were introduced to RAWM in 3 phases: adaptation (1 day), training (4 days), and probe (1 day). Under adaptation conditions, the mice were allowed to immerse into the pool and move freely for 2–3 min to adapt to the RAWM milieu. During the training, the mice were tested 5 times a day at an interval of 30 sec. During this period, the mice were given 60 sec to find the hidden platform. If the mice could not find the platform, they would be guided to the platform manually. In the probe phase, the platform was removed and the mice became free from the same area into the water and allowed to swim for 60 sec to find the platform site. The delay time to reach the platform location (sec), the time spent in the goal area (sec), and the proximity (cm) to the platform were recorded by a video camera and analyzed by an image tracking software (Noldus EthoVision XT7, the Netherlands).

***TUNEL assay***

In situ cell death detection Kit, Fluorescein (Cat. No. 11684795910, Roche Diagnostic GmbH, Applied Science, Germany) was used for TUNEL (Terminal deoxynucleotidyl transferase dUTP Nick-End Labeling) staining, and to identify and measure apoptotic cells by labeling the DNA strand breaks. After brain fixation and paraffinization, a coronal brain section (5-μm thick) was prepared for the TUNEL assay. Then tissues were deparaffinized: washed in phosphate buffer saline (PBS), immersed in proteinase K, permeabilized with penetrancation solution, washed in PBS, incubated in TUNEL solution, and finally washed with PBS. The TUNEL-positive cells were counted in 3 different visual fields for each section of the hippocampus (CA1, CA3, and DG), by a blinded assistant using fluorescence microscopy (×400, Olympus Corporation, Japan). Then data were analyzed in the Image J software package. The total number of nuclei stained by propidium iodide (PI, blue color) was calculated, and also the number of TUNEL-positive cells (green) was measured and expressed as a percentage of the total number of PI-stained nuclei.

***Cresyl violet (Nissl) staining***

Cresyl violet was used to identify neuronal damage and cell death. So coronal brain sections (5-µm thick) at the hippocampus level were prepared. After deparaffinization and hydration (in xylene, ethanol, and distilled water), the brain slices were stained with 0.1% cresyl violet solution (Sigma St. Louis, MO) for 10 min. Then sections were washed with distilled water, dehydrated in serially diluted ethanol, and mounted with Entellan. Neuronal cell damage and percent of cell death were measured by a light microscope and counted in CA1, CA3, and DG regions of the hippocampus. The number and percentage of damaged and dead nerve cells were measured by an optical microscope in CA1, CA3, and DG areas of the hippocampus.

***Western blotting***

As previously described, brain samples were used for measurement of TNFα, IL-1β, and Glyceraldehyde 3-phosphate dehydrogenase (GAPDH, ab181602, UK) by the Western blot method. The protein portion of the samples was extracted after tissue lysis in RIPA buffer and protease inhibitor cocktail. The concentration of proteins was measured by the Bradford method and then transferred to PVDF membranes (Roth) for 80 min at 80 V (Bio-Rad). Polyacrylamide gel electrophoresis (Bio-Rad) via 4–20% gradient polyacrylamide gels (contain 0.1% sodium dodecyl sulfate for ~2 hr at 95 V) were used for protein separation. After blocking with 5% skim milk in Tris-buffered saline and Tween 20(TBST) with pH 7.6, the membranes were incubated with primary antibodies against TNF-α (sc-133192; Santa Cruz, USA), IL-1β (orb382131; Biorbyt, UK), and GAPDH at 4 °C overnight. In the next step, the membranes with the appropriate horseradish peroxidase-conjugated secondary anti-rabbit antibodies (HRP, 1:5000, ab6721, UK), were incubated at room temperature for 2 hr. Then samples were washed 3 times for 10 min by TBST. Afterward, chromogenic substrate was added to the membranes and protein bands were detected using DAB (3, 3’-diaminobenzidine). Image J software was used to record and analyze substrate and membrane images. Finally, GAPDH was used for normalization. 

***Statistical analyses***

The results are presented as the mean±SEM and a *P-value* <0.05 was accepted as the statistically signiﬁcant diﬀerence. Because the data was normal, one-way analysis of variance (ANOVA) was used to compare the groups, which was followed by *post hoc* Holm-Sidak test. 

## Results

***Pretreatment of intranasal oxytocin improves neurological function***

**Figure 1:**
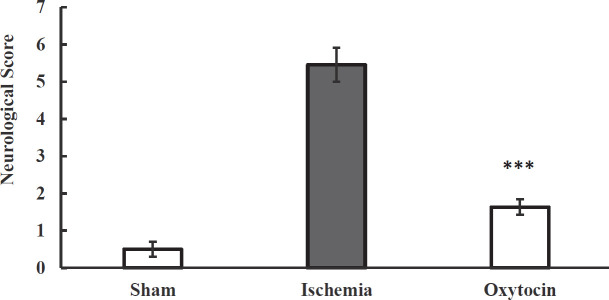
Pretreatment effect of intranasal oxytocin (8 IU/per mouse) on neurological disorder scores in the sham, ischemic, and oxytocin groups. ***P<0.001, compared with the ischemic group

Cerebral ischemia-induced by BCCAO for 20 min and blood flow reperfusion for 24 hr were associated with severe motor and sensory impairment in ischemic mice compared with sham groups. Pretreatment with oxt (8 IU/per mouse) for one week significantly improved these changes in the treatment group compared with the ischemic mice (*P*<0.001, Figure 1).

***Pretreatment of intranasal oxytocin improves spatial learning and memory***

**Figure 2:**
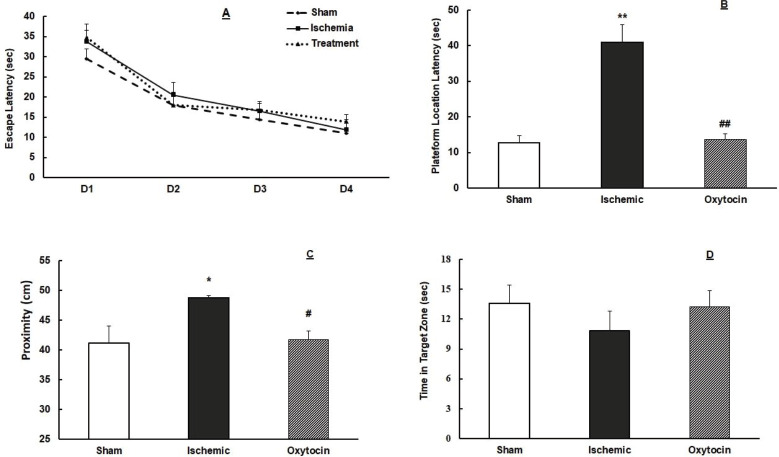
Pretreatment effect of intranasal oxytocin (8 IU/per mouse) on spatial learning and memory. Escape latency (sec) in 4-day (D4) training in the radial arm water maze (A), the proximity (cm) to the platform (B), time (sec) to find the platform location (C), and time spent in the target zone (D) in sham, ischemic, and oxytocin groups. * *P*<0.05, ** *P*<0.01 compared with sham. # *P*<0.05, ## *P*<0.01 compared with ischemic groups

 Spatial learning and memory during the 4-day training in RAWM showed the time to find the hidden platform is reduced gradually in mice. There was no significant difference between the groups in the training phase (Figure 2A). Nevertheless, in the probe trials, induction of global ischemia increased the time (sec) to find the location of the platform (*P*<0.01) and the proximity (*P*<0.05) to the platform compared with sham groups (Figures 2B-D). Pretreatment with oxytocin significantly reduced the time to find the place of the platform (*P*<0.01) and proximity (*P*<0.05) to the platform (Figures 2B-D). 

***Pretreatment of intranasal oxytocin reduces brain damage: cresyl violet staining***

**Figure 3:**
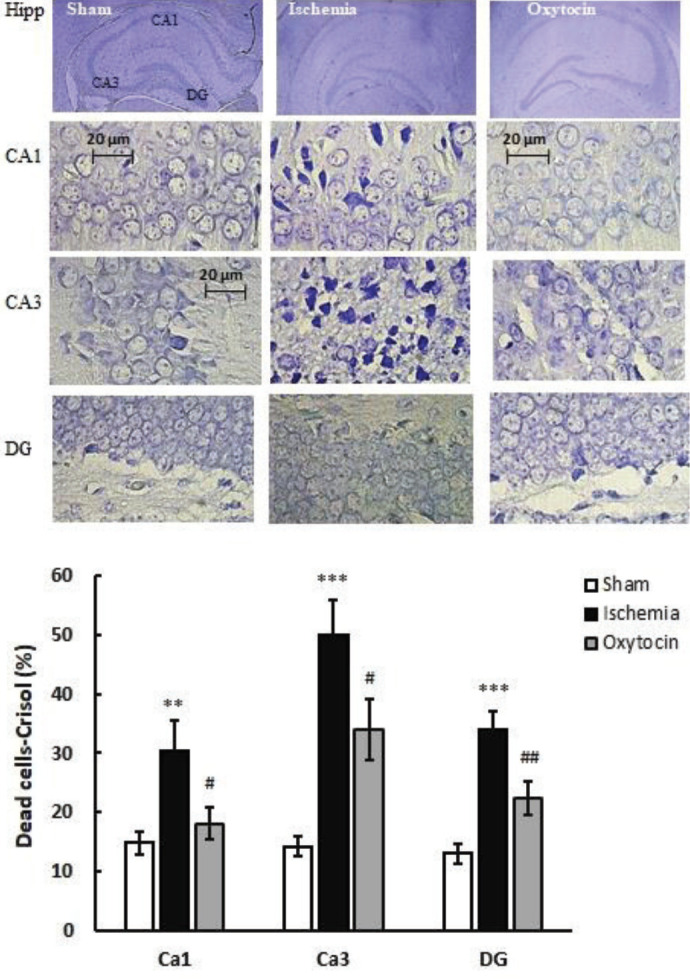
The photomicrograph (top) shows the pretreatment effect of intranasal oxytocin (8 IU/per mouse) on the percent of dead cells in CA1, CA3, and DG in sham, ischemic, and oxytocin groups identified in cresyl violet staining. A quantitative analysis of the percentage of dead cells is also shown in the diagram (below). ***P<0.001 and **P<0.01 compared with sham. ## *P*<0.01 and # *P*<0.05 compared with ischemic groups

Cerebral ischemia-induced by BCCAO resulted in severe brain damage and cell death. Cresyl violet staining of the hippocampus in the ischemic group showed neuronal cell death in CA1 (30.3%), CA3 (50%), and DG (33.98%). Oxt (8 IU/per mouse) significantly reduced neuronal cell death in CA1, CA3, and DG by17.9, 33.9, and 22.3%, respectively (Figures 3 A & B). 

***Pretreatment of intranasal oxytocin reduces apoptosis in the hippocampus: TUNEL staining***

**Figure 4:**
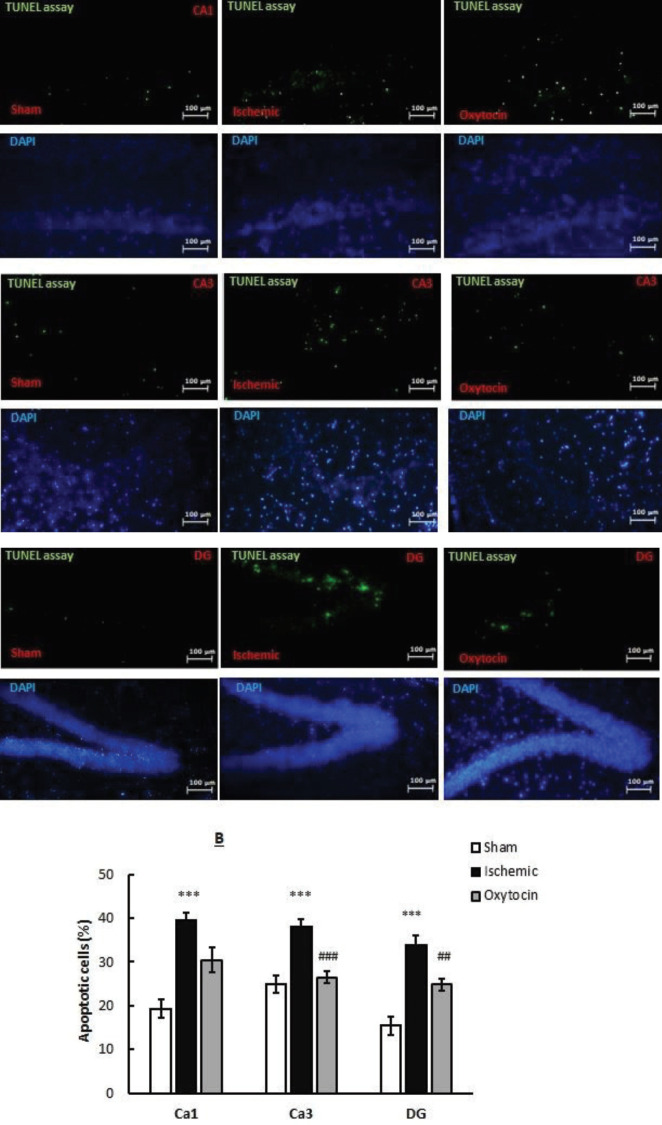
Photomicrograph (top) shows the effect of intranasal intranasal oxytocin (8 IU per mouse) on the number of apoptotic cells in CA1, CA3, and DG in sham (A), ischemic (B), and oxytocin (C) groups identified in TUNEL staining. A quantitative analysis of the percentage of TUNEL-positive cells is also shown in the diagram (below). TUNEL-positive cells (green) were expressed as a percentage of the total number of DAPI-stained nuclei (blue) (400×fluorescent microscope). ***P<0.001 compared with sham. ## *P*<0.01 and # *P*<0.05 compared with ischemic groups

Cerebral ischemia induction resulted in a significant increase in the apoptotic cells in the hippocampus compared with the sham group (*P*<0.001, Figures 4B). Immunohistochemical measurements in CA1, CA3, and DG of the ischemic mice showed that positive TUNEL cells increased by 37.9, 38, and 33.9%, respectively. Oxt (8 IU/per mouse) significantly reduced positive TUNEL cells (apoptotic cells) in CA1, CA3, and DG by 31.98, 26.6, and 24.8%, respectively. 

***Pretreatment of intranasal oxytocin reduces TNFɑ and IL-1β protein level in the hippocampus***

**Figure 5:**
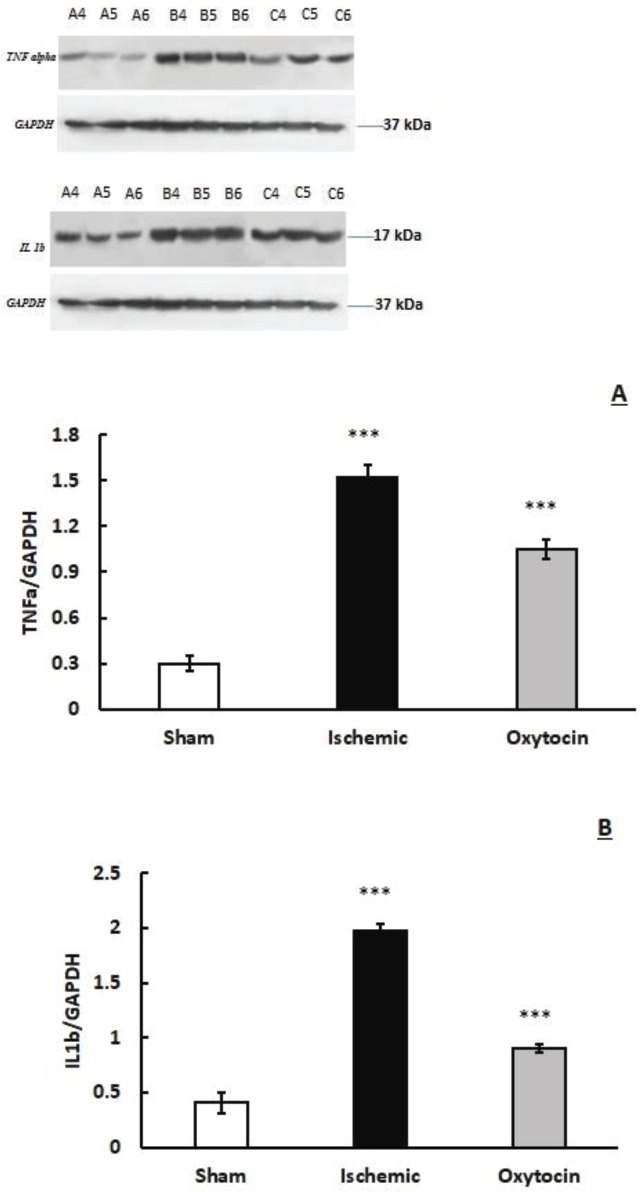
Photograph shows the effect of intranasal intranasal oxytocin (8 IU/per mouse) on levels of TNF-α and IL-1β (Western blotting) in sham, ischemic, and oxytocin groups. Quantitative analysis shows (A) TNF-α/GAPDH and (B), IL-1β/GAPDH in the brain tissue. ***P<0.001 compared with the sham group. ### *P*<0.001 compared with the ischemic group

Cerebral ischemia induction resulted in a huge increase in the levels of TNFɑ and IL-1β protein in the hippocampus (*P*<0.01, Figures 5A-B). Oxytocin (8 IU/ per mouse) reduced TNFɑ and IL-1β protein levels compared with ischemic mice to 30.8 and 54.5%, respectively. 

## Discussion

The prophylactic effect of oxt was investigated in a general cerebral ischemic model in mice. Cerebral ischemia was induced for 20 min by BCCAO and then blood flow was restored for 24 hr. Cresyl violet and TUNEL staining showed extensive cell death and apoptosis in CA1, CA3, and DG of the hippocampus in ischemic mice. These injuries increased the score of the neurological disorder to about 6, which is consistent with moderate sensory-motor impairment. In addition, learning and spatial memory were significantly impaired. On the other hand, pretreatment with oxt for a week reduces neuronal death and apoptosis in C1, C3, and DG of the hippocampus. In agreement with our results, several animal studies have shown that exogenous oxytocin reduces ischemic damage to ovarian, liver, stomach, and heart tissues (14, 15). Our recent study also shows oxytocin reduces brain injury in the early stages of stroke by inhibiting apoptotic and nuclear factor-kappa B (NF-κB) signaling pathways. In addition, oxytocin increased the expression of vascular endothelial growth factor (VEGF), aquaporin-4 (AQP4), and brain-derived neurotrophic factor (BDNF) proteins and reduced the blood-brain barrier (BBB) leakage (16). Oxytocin can also protect the brain against stroke induced damage by reducing the neuroinflammatory and oxidative stress pathways (17). Also, oxytocin prevents apoptosis, oxidative stress, and inflammatory cytokines in various tissues (10, 18). 

In this study, neuronal damage in the basal ganglia and sensory-motor cortex was not examined. But improving neurological disorders in treated mice suggests that oxytocin may also reduce damage or prevent the spread of damage to these areas. Also, our results showed an improvement in spatial memory in the treatment group. In agreement with our results, Dayi *et al.* have reported impaired hippocampal learning and memory due to chronic stress can be improved by oxt (19).

TNFα is a pro-inflammatory cytokine produced by many cells, especially by macrophages and microglia (20). It is one of the leading causes of inflammatory responses and is thought to worsen stroke status (21). The levels of TNFα in blood circulation and cerebrospinal fluid rise rapidly following a stroke, and stroke lesion size has a positive correlation with TNFα levels (22). Various studies have shown an association between TNFα and stroke injury (20). Similarly, TNFα has been extensively increased in this study, which may mediate some brain damages. Pretreatment with oxt reduces TNFα by reducing NF-κB (15) or other mechanisms, which are associated with improved neurological disorders and spatial memory.

Several regulatory molecules are involved in inflammation, but the IL-1 family has a key central role in this regard (23). Interleukin-1 is a cytokine that is involved in brain ischemic damage in rodents (24). Brain ischemia caused a severe increase in IL-1 levels in this study. Pretreatment with oxytocin significantly reduced ischemic damage apparently by a reduction in levels of IL-1. Similarly, IL-1ra, a highly selective IL-1 receptor antagonist, reduces cell death when injected peripherally in transient cerebral ischemia (25, 26). Also, recombinant human IL-1ra (rhIL-1ra) has beneficial effects in human acute stroke and has been suggested as a potential new therapeutic agent for acute stroke (27).

Nuclear factor-kappa B (NF-κB) is a transcription factor involved in the regulation of the inflammatory process, oxidative stress, immunity, and apoptosis (28, 29). The expression of more than 35 genes, including TNF and IL, is regulated by NF-κBI, and in turn, TNF and IL induce NF-κB activity. Therefore, NF-κB inhibitors are used to treat diseases mediated by NF-κB (29). Cerebral ischemia also increased NF-κB, which causes the expression of IL-1, IL-6, and TNF genes that participate in brain damage (30, 31). Our results show that pretreatment with oxytocin reduced IL-1β and TNF-α expression, probably by inhibiting NF-κB in the brain tissue as it has been reported recently (16). Additionally, several *in vitro* and *in vivo* studies have shown that oxytocin has anti-inflammatory activity by inhibiting the expressions of NF-κB and proinflammatory cytokines (32), which confirms our finding.

## Conclusion

Oxytocin reduced cell death and apoptosis caused by cerebral ischemia, which coincided with improved neural function and spatial memory. Oxytocin also reduced inflammatory mediators that were severely increased during ischemia. Oxytocin appears to have therapeutic effects by reducing inflammatory factors and neural death. Therefore, pretreatment with oxytocin may be helpful in people who are prone to stroke.
